# Knowledge, attitudes and practices towards fetal alcohol spectrum disorder among lawyers in New Zealand

**DOI:** 10.1080/13218719.2023.2243310

**Published:** 2024-01-08

**Authors:** Joanna Ting Wai Chu, Holly Wilson, Jessica C. McCormack, Valerie McGinn, Warren Brookbanks, Chris Bullen

**Affiliations:** a National Institute for Health Innovation, School of Population Health, The University of Auckland, Auckland, New Zealand; b Food Science, University of Otago, Dunedin, New Zealand; c The FASD Centre Aotearoa, Auckland, New Zealand; d Criminal Law and Justice Studies, Auckland University of Technology, Auckland, New Zealand

**Keywords:** attitudes, fetal alcohol, justice professionals, knowledge, lawyers, practices, survey

## Abstract

Fetal alcohol spectrum disorder (FASD) is a developmental disability that can cause difficulties with communication, emotional regulation and executive function, making people with FASD vulnerable to adverse involvement within the criminal justice system. Justice professionals’ knowledge and attitudes of FASD is critical to identifying appropriate responses, management and sentencing in the justice system. This research aims to understand the FASD knowledge, attitudes and practices among lawyers working in the justice sector in Aotearoa, New Zealand. We conducted an online survey on the awareness, knowledge and beliefs of FASD, experience and professional practice with FASD of justice professionals working in the justice sector in New Zealand. Of the 56 participants, most were lawyers. All participants were aware of FASD but had gaps in their knowledge and few felt well prepared to support someone with FASD. There is a need to develop policies, training and support for lawyers, around FASD.

## Introduction

Fetal alcohol spectrum disorder (FASD) is a diagnostic term that describes the neurological and physical effects of prenatal alcohol exposure (PAE; Cook et al., 2016) and is one of the most common forms of neurodevelopmental disorder (Flannigan et al., 2021). The global prevalence of FASD is estimated at 7.7 per 1000 births, but estimates vary considerably across countries (Lange et al., 2017). In Aotearoa, New Zealand, the Ministry of Health estimates that of every 100 live births, 3 to 5 are affected by alcohol (Ministry of Health, 2022). However, this is likely to underestimate the actual number of those living with FASD due to difficulties accessing a formal diagnosis (Coons et al., 2018; Mukherjee et al., 2013).

Symptoms of FASD include restricted growth, diminished neurological and cognitive functioning, characteristic facial features and behavioural problems. These symptoms result in difficulties across various neurodevelopmental domains for people with FASD, such as difficulties with memory, cognition, language, executive function, social skills and attention, which are common (Cook et al., 2016). These difficulties affect an individual’s functional abilities and can lead to early school failure, poor mental health, substance misuse and engagement with the justice system (Petrenko et al., 2014; Streissguth et al., 2004). An analysis of the Canadian FASD database that collates information from 26 FASD diagnostic clinics throughout Canada found that 30% of youth and adults diagnosed with FASD were in current conflict with the law, compared with less than 2% in the general population (McLachlan et al., 2020). High incarceration rates have also been observed, for example, 36% of youth in a detention centre in Western Australia met diagnostic criteria for FASD (Bower et al., 2018) and 23.3% of youth in a forensic mental health facility in Canada (Fast et al., 1999).

Individuals with FASD may face profound challenges within the criminal justice system, whether as an accused individual, witness or victim. While the manifestation of FASD impairments varies considerably from person to person, common features include difficulties decoding non-verbal cues, understanding non-literal language and accurately recalling information (Fast & Conry, 2009; Hand et al., 2016). These aspects of FASD render individuals with the disorder vulnerable to poor outcomes in the justice system as they may not be able to participate effectively in negotiation or dispute settings, such as in a court of law, and to effectively engage with the justice system to get fair outcomes (Brookbanks et al., 2022; Reid et al., 2020). In addition, a departure from daily routine and lack of control of the situation can be significantly distressing for individuals with FASD.

In some instances, court proceedings have considered the effects of FASD and can affect the sentencing outcomes, provided that they are able to access diagnosis or assessment to effectively support people with FASD within the justice sector (Reid et al., 2020). FASD can be a largely invisible disability, with less than 10% showing distinctive facial features (Andrew, 2010). Understanding and awareness of the lifelong brain impairment, cognitive deficits and the ongoing difficulties experienced by those with FASD are, therefore, critically important for professionals in the justice sector to properly represent and judge individuals with FASD. Without this understanding and support, people with FASD can often face negative experiences and potentially unfair interactions with and treatment in justice settings.

Despite the potential implications of FASD for people involved in the courts, few studies have examined the knowledge, attitudes and practices (KAP) of justice professionals. A recent systematic review revealed only eight studies that have surveyed KAP in justice settings (McCormack et al., 2022). Professional groups sampled included forensic mental health professionals, judges and judicial officers, lawyers, police officers and youth custodial officers. A 2013 survey of justice professionals (*n* = 427) in Western Australia found that all lawyers (88% of the participants) reported being aware of FASD (Mutch et al., 2013). However, 44% of lawyers reported being aware of FASD but not how it affects children and adults. Respondents identified a lack of knowledge and access to diagnosis and health professionals as challenges to dealing with and ensuring fair justice outcomes for an individual with FASD (Mutch et al., 2013). A 2008 survey of judges and prosecutors (*n* = 39) in Canada showed that most had experience working with an individual with FASD, and many indicated that an FASD diagnosis would impact their practice (Cox et al., 2008). However, less than 40% of judges and prosecutors felt prepared to deal with FASD cases or work with people with FASD (Cox et al., 2008).

In New Zealand, there has been limited research on KAP of FASD in the justice sector. A qualitative study with 11 participants with experience working with FASD and the justice sector showed a lack of knowledge and understanding of FASD within the justice sector in New Zealand. Respondents identified the need for professional training and support for those working in the justice sector (Gibbs, 2022). Therefore, there is a need to explore the KAP of those in the justice sector to identify training and knowledge gaps and, ultimately, improve the support and experiences of those with FASD. This study aims to understand the knowledge of FASD, attitudes and practices (KAP) of lawyers working in the justice sector in New Zealand.

## Method

### Design

We conducted an online KAP survey of lawyers working in the justice profession in New Zealand. The survey consisted of multi-choice, true–false and open-ended questions and took 5–10 min to complete. The survey was adapted from a survey we conducted of New Zealand educators (Chu et al., 2022), which was modelled on previous surveys of health and justice professionals (Cox et al., 2008; Mutch et al., 2013; Payne et al., 2011; Would, 2009). We describe the development of that survey in full in Chu et al. (2022). We adapted the survey questions in Chu et al. (2022) to describe the work of people in the justice sector and sought feedback from sector workers to ensure that the wording was appropriate. The survey measured four domains:

*Awareness of FASD* was measured with three items, to measure participants’ awareness level and where they learned about FASD. A sample item is ‘*Had you heard about FASD before this survey?’*

*Knowledge and beliefs about FASD* were measured with 13 items. We measured participants’ knowledge of domains affected by FASD through six true–false statements. Beliefs about FASD were measured on six true–false statements; these items measured several beliefs, including, but not limited to, stigma and the impact of diagnosis. A sample item is *‘Diagnosis of FASD may lead to an individual or their family being stigmatised*.*’*

*The impact of FASD on professional practice and experience with FASD* was measured with six items. These items measured beliefs on the prevalence of FASD, participants’ experience with FASD in their work and how FASD could impact their professionals’ practice. An example item is ‘*If someone presented in court (i.e.* *client, defendant, witness, complainant) was known* *to have FASD, would that change your practices of behaviour towards them?’*

*Training and information needs relating to FASD* were measured with six items. These items measured the training and support that lawyers felt they need to support to work with people with FASD. A sample question is ‘*What do you think is the most important information that you need to know about FASD for your work?’*

The survey also collected demographic information, location (i.e. rural, urban or city centre), role in the sector, types of work setting and years working in the sector.

### Study population

Participants were eligible to participate in the survey if they were aged ≥18 years, self-identified as a justice professional, resided in New Zealand, could read and speak English, were currently employed in New Zealand and had contact with clients in their work. In New Zealand in 2021 there were 15,554 lawyers with a current practising certificate, approximately half were male (46.0%), most of the lawyers identified as European (76.7%), and 6.9% identified as Māori. The lawyers with a current practising certificate are working in several aspects of law; the most common areas include company and commercial law, property and civil law, and the least common areas include media law, real estate and accident compensation (Barnett et al., 2021).

### Recruitment

We recruited participants over 5 months from April 2022 to September 2022 through mailing lists, social media platforms (Facebook, Twitter) and e-newsletters to professional groups (i.e. New Zealand Law Society, The Māori Law Society – Te Hunga Rōia Māori o Aotearoa). The Public Defence Service – a unit that operates independently within the Ministry of Justice – was contacted, and recruitment material was distributed via their e-newsletter, Law Points. Social medial links were shared through provider and advocacy networks, including the New Zealand FASD Care Action Network (FASD-CAN) and Australia New Zealand FASD Clinical Network (ANZFASD-CN). Personal communications and word of mouth were used to recruit judges.

We obtained consent from participants on the aims and focus of the survey via an online participant information sheet before starting the survey. Completing the survey was equivalent to consent for their answers to be used in the research.

### Analysis

We collected and managed all study data via an online survey created in a REDCap database (Harris et al., 2019) hosted by the University of Auckland and exported to SPSS Version 28 for analysis (IBM Corp, 2017). We summarized continuous variables as frequencies, means and standard deviations (*SD*s), medians and interquartile range. We summarized categorical variables as percentages. We exported open-ended answers to NVivo (QSR International Pty Ltd, 2020) for analysis using reflexive thematic analysis methods (Braun & Clarke, 2022). The first phase of analysis was familiarization, followed by coding open-ended responses using semantic codes (Terry & Hayfield, 2020). Once all data were coded, we built thematic maps to identify clusters around central themes with shared meaning, which H.W. and J.C. then reviewed.

## Results

In total, 84 people responded to the invitation to participate. We excluded 28 responses from people who did not complete the survey or did not answer the screening question. Our final sample consisted of 56 participants (Table 1). It should be noted that although we intended to examine lawyers’ and judges’ knowledge, attitudes and practices of FASD, none of the participants identified as judges despite recruitment efforts.

**Table 1.** Demographics of participants showing count and percentage.

Demographics	Participants	
	*N*	%
Gender		
Male	11	19.6
Female	45	80.4
Ethnicity		
NZ European	39	69.6
Māori	5	8.9
Samoan	1	1.9
Indian	2	3.6
Prefer not to say	9	16.1
Location		
Suburbs	26	46.4
Inner city	19	33.9
Rural	11	19.6
Role in justice sector		
Lawyer	51	91.0
Other	4	7.1
Prefer not to say	1	1.8
Area of practice in justice sector		
Criminal law	17	30.4
Family law	16	28.5
Corporate or community law	4	7.1
Property law	2	3.6
Tort law	1	1.8
Public law/ Te Triti o Waitangi	2	3.6
Employment law	3	5.4
Other	10	17.9
Prefer not to say	1	1.8
Years employed in the justice sector		
0–1 years	0	0
1–2 years	7	12.5
3–4 years	10	17.9
5–10 years	7	12.5
11 or more years	32	57.1

Most participants who responded to the survey were lawyers (*n* = 51, 91.0%). Of the lawyers, most worked in criminal law (*n* = 17, 30.9%) or family law (*n* = 16, 29.1%). Most participants had been practising law for 11 or more years (57.1%), and no participants had been practising for less than 1 year.

Most participants identified as female (*n* = 45, 80.4%), New Zealand European (83.0%) and lived in lived in suburban (*n* = 26, 46.4%) areas.

### Awareness and knowledge of FASD

Almost all participants (98.2%) reported they were aware of FASD prior to taking part in this study. The most common sources of information about FASD were professional training (44.6%), mainstream media (44.6%), friends or family (42.9%), and professional resources (30.4%).

When asked to rate their knowledge of FASD, most participants reported a basic understanding of FASD and its effects (66.2%). While 23.2% of participants reported having a good understanding of FASD and its effects, only 3.6% had a good understanding of FASD and its effects on the criminal justice system. Around 5.4% reported being aware of FASD and its effects. Only one participant reported having never heard of FASD before. Participants were given a list of characteristics and were asked to identify three features required for diagnosing FASD (central nervous system abnormality, neurological impairments and exposure to alcohol during pregnancy; Cook et al., 2016). Most participants correctly identified two diagnostic features (67.9%), but few identified all three (19.6%). Most participants correctly identified exposure to alcohol during pregnancy (91.1%) and neurological impairment (92.9%). However, only 23.2% correctly reported central nervous system abnormality.

We asked participants to estimate how common they believed FASD to be in New Zealand. The Ministry of Health’s estimate of FASD in New Zealand has recently been updated from 1–3% to 3–5% (Ministry of Health, 2022). We, therefore, treated both 1 in 50 (2%) and 1 in 20 (5%) as correct responses. Only 29.6% of participants correctly estimated the prevalence of FASD in New Zealand (18.5% choosing 1 in 50 people and 11.1% choosing 1 in 20 people), while most participants (64.8%) underestimated the prevalence of FASD. We asked participants to estimate how common FASD is likely in people involved in the justice system, including incarcerated adults, children in the care of youth justice, arrestees and victims of crime. A majority (85.2%) of participants believe that at least 1 in 50 people involved in the justice system were affected by FASD (14.8% choosing 1 in 20 and 16.7% choosing 1 in 10 people), with 42.6% of participants believing it was 1 in 5 people.

### Attitudes and beliefs towards FASD

**Figure 1.**
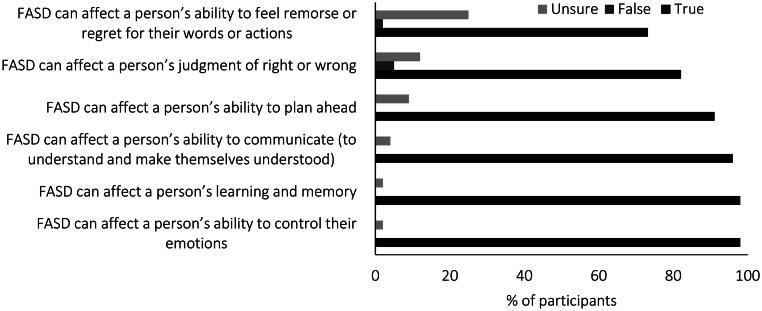
Percentage of participants responding to each domain affected by fetal alcohol spectrum disorder (FASD).

Participants reported on the domains they thought would be affected by FASD in true–false statements (see Figure 1). Most participants (more than 90%) correctly identified that FASD could affect a person’s learning, emotional control and ability to communicate and plan. However, many participants believed that FASD could affect a person’s ability to feel remorse (73%) and judge right and wrong (82%).

**Figure 2.**
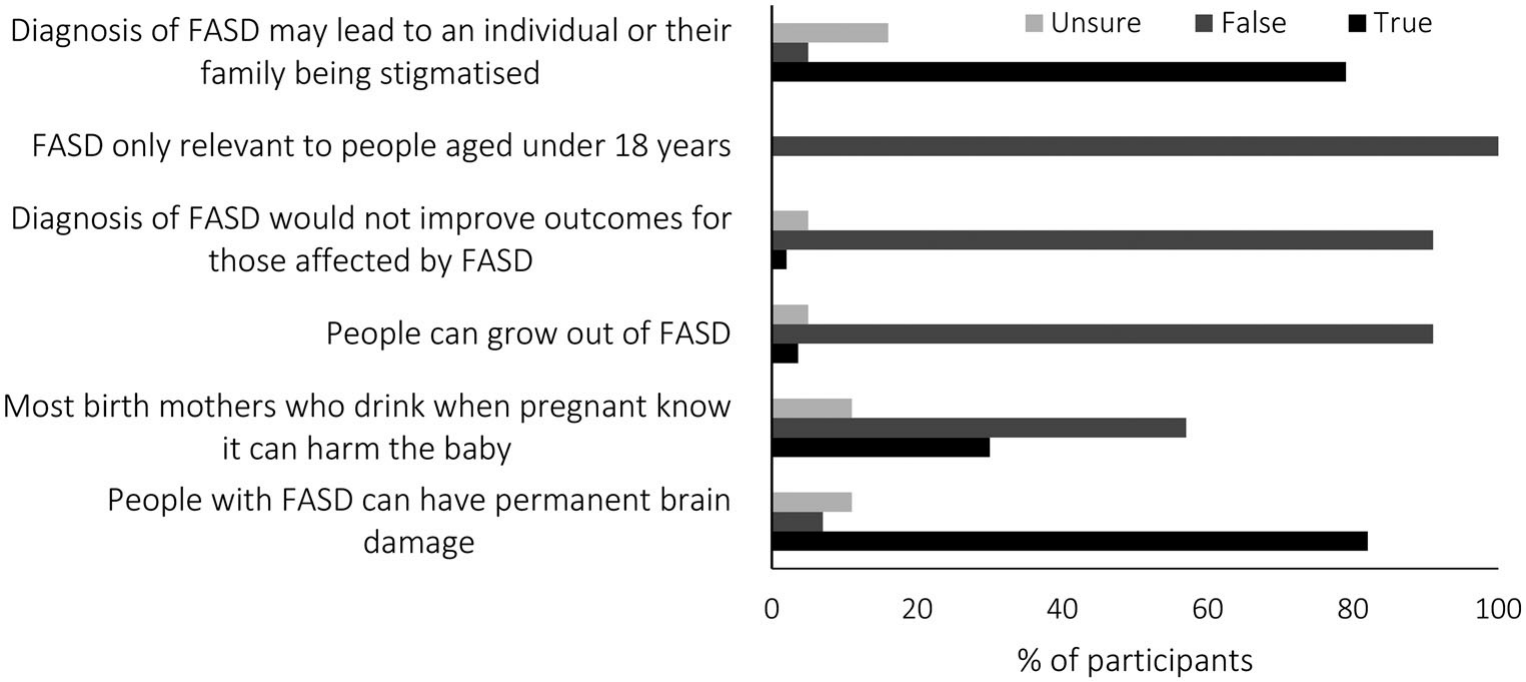
Percentage of participants reporting beliefs about fetal alcohol spectrum disorder (FASD).

We assessed general beliefs about FASD through a series of true–false statements (Figure 2). Most participants correctly reported that FASD could result in permanent brain damage and correctly rejected that people could grow out of FASD and that FASD is only relevant to people under 18. Similarly, most participants rejected that a diagnosis would not improve outcomes. Participants’ responses varied for the belief that birth mothers were aware of the harm of drinking while pregnant, with 57.1% of participants choosing false and 30% choosing true. Most participants (78.6%) felt that a diagnosis of FASD could lead to stigma, with 5.4% choosing false and 16.1% unsure.

### Experiences and practices when encountering a person with FASD

Most participants reported that FASD was highly relevant (44.6%) or relevant (26.8%) to their work in the justice sector, and 12.5% of people thought that FASD was highly irrelevant or irrelevant (3.6%). Over 50% of participants had or suspected they had encountered FASD while working in the justice sector (25.5% suspected and 32.7% diagnosed), and only 7.3% of participants had not encountered someone with FASD in their work. Most participants reported they would change their practices (90.9%) if they knew someone had FASD. When asked to explain their answer (*n* = 45), most participants recognized the need for different communication strategies to engage with their clients. Participants gave specific examples such as using simple language, spending more time explaining and repeating concepts to ensure understanding, and requesting communication assistance. For those who would not change their practices or behaviour, participants reported that they were already responsive to individual needs and differences. Most participants (88.9%) believed that knowing a defendant had FASD would affect sentencing. Many participants reported that failing to account for FASD would be contrary to the purpose/aims/goals of the criminal justice system, specifically that sentencing should be *‘rehabilitative rather than punitive’*. Participants also noted the importance of sentencing being tailored to meet individual needs and that obtaining a neuropsychological report before sentencing would be helpful to accommodate such needs. For participants that did not believe a patient with FASD would affect their sentencing, a typical response was *‘not sure’*. One respondent stated that *‘many factors contribute to behaviour and diagnosis of FASD should not absolve someone of their responsibility for their actions’*.

Only three participants (5.4%) felt very prepared to support someone with FASD in the justice system, most participants felt moderately prepared (32.1%) or somewhat unprepared (39.3%), and 23.2% felt not at all prepared.

### Education and training related to FASD

Over half of the participants (58.9%) had not received training about FASD or other neurodevelopmental disorders. Only 33.9% had any FASD-related training, and 8.9% had training for other neurological or neurodevelopmental disorders in the last 5 years. Likewise, few participants were aware (7.3%) and had used (5.5%) the FASD training resources provided by Te Pou – a Ministry of Health-funded workforce development provider.

When asked what information would help prepare them to work with individuals with FASD within the justice system, 44 participants highlighted the need for more training in general and noted that *‘there isn’t anywhere near enough training available through the professional bodies’*. Resources on recognizing FASD, the effect of FASD, communication strategies with the client and family members and how best to support clients with FASD through the legal system were common information sought by participants. Participants also noted the need to have specialist reports detailing the diagnosis and impairment of the individual. Participants also noted that access to legal aid funding and specialist support (e.g. health professionals and communication assistants) was an issue.

A few participants highlighted the need to increase FASD knowledge among other professionals in the wider justice sector. For example, one participant expressed that they *‘often have to convince people that FASD is real and has serious impacts on person affected’*. Participants believed that most resources options, including written material (75%), protocols and guidelines (73.8%), training workshops (71.8%), online resources (73.8%), referral resources (64.3%) and information for inmates and families (50%) would be helpful for their work in the justice sector. Similarly, participants believed that information on guidelines to support people with FASD (82.1%), contact information for organizations that support individuals living with FASD (60.7%) and referral information for diagnosis was most important in their work (55.4%).

Two themes emerged when asked about potential challenges for working with individuals living with FASD. The first was around having the appropriate knowledge and skills for working with people with FASD – personally and with other professionals working in the justice system. Participants noted that the concern for them personally was not knowing that their client might have FASD, leading to *‘dealing with the client in a way that is inappropriate to their situation’*. Several participants further commented on the *‘minimal understanding of FASD in the justice system’*, *‘the need for FASD to be recognized in the wider justice system’* and *‘practical guidelines to be put in place for lawyers and other justice sector stakeholders’*. One participant felt that *‘there seems to be very little compassion for this deficit that makes fighting for its recognition necessary at every stage of the process, from engagement with forensics, approval for funding from legal aid, recognition of the deficit at sentencing’*.

Another theme concerns the difficulty in getting an assessment or diagnosis conducted for the individual. For example, participants noted the difficulty in getting an assessment done promptly, the lack of support for the referral process, the lack of funding to obtain an assessment and the barriers around convincing the client and other stakeholders to be diagnosed due to stigma. Several participants also noted the lack of support post diagnosis. For example, one participant questioned, *‘there is no Ministry of Health funding* *–* *what is the benefit of getting a diagnosis long term? It may help with legal proceedings (culpability and sentencing), but where next for that person?’*.

## Discussion

This study is the first in New Zealand to survey lawyers in the justice sector about their knowledge, attitudes and practices about FASD. We found that while lawyers working in the justice sector in New Zealand have some knowledge about FASD, gaps in knowledge and inaccurate beliefs are common. Most participants felt unprepared to support a person with FASD to navigate the justice system. Consistent with a previous survey where 40% of judges and prosecutors felt prepared to support someone with FASD (Cox et al., 2008), around 40% of our respondents felt prepared to support people with FASD, with only around 5% feeling very prepared. This lack of knowledge about FASD is similar to findings of our previous survey with education and social and community workers (Chu et al., 2022; McCormack et al., 2023) and surveys with justice professionals (Cox et al., 2008; Mutch et al., 2013).

Recognizing that an individual may have FASD and referring them appropriately require professionals to have adequate awareness of and knowledge about the disability. It is often the responsibility of lawyers or counsel to raise a possible neuro-disability in the legal process and to ensure that relevant medical evidence is sought to ensure a fair trial. This is especially important in the case of FASD, where the disability may not be visible (Andrew, 2010) or unrecognized until the legal process. Our respondents recognized the need for individuals engaging with the justice system to be referred for neurodevelopmental assessment to identify impairments and obtain complete information about their diagnosis so that degree of culpability can be considered and an effective response formulated. However, many respondents reported only a basic understanding of FASD, with several participants concerned about not being able to recognize the signs of FASD and alerting relevant individuals of the need for referral. While New Zealand does not currently have clear diagnostic guidelines of FASD, the Ministry of Health specifies that a diagnosis of FASD is evidenced by PAE and impairment in at least three domains (Ministry of Health, 2022). The Canadian guidelines specify the 10 neurocognitive domains, such as language, cognition and social skills (Cook et al., 2016); therefore, difficulties with these specific domains or everyday life, such as in school or employment, could be indicative of FASD. There is a risk that an individual with FASD may navigate the youth justice system without recognition of their disability.

People with FASD can experience varied impairments stemming from PAE. These impairments can vary for individuals living with FASD and can manifest in different effects. Respondents in this survey correctly identified several of the common impairments that people with FASD can experience (Cook et al., 2016), including learning, emotional control and the abilities to communicate and plan ahead. Most participants also felt FASD could impair people’s ability to judge right and wrong, and ability to show remorse. These beliefs are largely consistent with the beliefs of educators (Chu et al., 2022) and those employed in the social and community sector (McCormack et al., 2023) in New Zealand. Judgment and remorse are not included in the neurocognitive domains that FASD can affect (Cook et al., 2016). However, judgment and remorse are higher order thinking processes included under the executive function domain. PAE can affect a person’s executive function (Cook et al., 2016), which is vital for academic achievement, social skills, everyday life skills and development (Brown & Reynolds, 2021). Impairments in executive functioning can impair the ability of people with FASD to plan ahead and comprehend consequences of actions and the feelings of others (Brown & Reynolds, 2021), which could impair the ability to show remorse for people with FASD (Wartnik et al., 2021). This may have negative implications on court proceedings and sentencing for those with FASD (Wartnik et al., 2021), potentially leading to harsher sentencing or denial of probation and extended incarceration.

Almost a third of the participants believed that most birth mothers who drink when pregnant are aware of the potential harm to the baby, suggesting that participants may hold stigmatizing beliefs about women who gave birth to children prenatally exposed to alcohol. Previous research has found that the general public can hold negative attitudes towards mothers of children with PAE (Roozen et al., 2022). For those affected by FASD, stigma can be a barrier to seeking a diagnosis (Mukherjee et al., 2013). Some lawyers in this survey reported that lack of understanding of FASD and stigmatizing beliefs amongst those working in the justice sector were challenging for working with those with FASD. In some situations, lawyers had to advocate for the recognition and fair response to FASD in the justice sector, suggesting that stigmatizing beliefs and a lack of understanding of the effects of FASD could hamper efforts for fair and equitable treatment in the New Zealand justice sector.

Courts in New Zealand have accepted FASD as a mitigating factor when considering applying suitable sanctions for lawbreaking (Gibbs & Sherwood, 2017). However, despite this, those working with people with FASD, many in the justice sector in New Zealand, feel unprepared to support someone with FASD and help them navigate the justice system. Although most participants believed they would change their behaviour if they knew someone had FASD, to be restorative, they expressed difficulty navigating within the system due to the lack of knowledge and policies, compounded by difficulty accessing support. Participants reported that there was little recognition of FASD in the justice system, so improving awareness of FASD within the justice may help in developing policies and practices that accommodate FASD within the courts. Without advances in recognition and understanding, there could be continued misrecognition and sanctions for those with FASD, such as the case of a Māori teenager who was wrongly convicted and imprisoned for 22 years (Freckelton, 2016).

Currently, FASD is not recognized in New Zealand as a funded disability, and only those with a low level of intellectual ability are eligible for disability support, excluding the majority of people with FASD (Disability Rights Commissioner & Children’s Commissioner, 2021). Accessing a diagnosis in New Zealand is hampered by a lack of diagnostic guidelines and experts to diagnose FASD (Gibbs & Sherwood, 2017), leaving people waiting for a long time to be assessed for diagnosis. Lawyers in this survey reported that accessing a timely diagnosis is challenging when helping people with FASD navigate the justice system. In the absence of a diagnosis, individuals with FASD are unlikely to receive the support needed to navigate the justice system, and there is a risk that FASD may not be taken into account in legal proceedings, leading to unjust outcomes. Lawyers who completed this survey also recognized that if people receive a diagnosis, they receive little support, so it is vital that people with FASD can access support to help them through the justice system and continue to support them after the legal proceedings.

Our findings highlight the significant gaps in knowledge and awareness of FASD in New Zealand and the need for professional development and training for lawyers and all justice professionals to ensure fair and just treatment for people with FASD in the justice system. Lawyers in this survey struggled to find training or resources to help them support someone with FASD and felt a range of resources would help them support someone with FASD. In contrast to other professional groups surveyed (Chu et al., 2022; McCormack et al., 2023), fewer lawyers reported professional or academic resources or colleagues as a source of information about FASD, and more reported informal sources, such as mainstream media and friends and family. A lack of training and support is a common issue among professionals working with people with FASD in New Zealand (Chu et al., 2022; McCormack et al., 2023) and internationally for those working in the justice sector (McCormack et al., 2022; Mutch et al., 2013), indicating the need for recognition, professional training and support to help professionally support those with FASD. Most lawyers who responded to this survey believed that training workshops, protocols and guidelines would support them to work with those with FASD. While there has been very little research into workforce development around FASD, a training workshops with professionals working in youth justice centres improved knowledge and attitudes towards those with FASD (Passmore et al., 2021). Comparatively, written resources provided to paediatricians did not significantly improve their knowledge and awareness of FASD (Payne et al., 2011). Therefore, potentially workshops throughout justice professional careers and policies to guide professionals could be the most effective at improving knowledge, attitudes and practices of FASD for justice professionals, ideally incorporated into tertiary education programmes.

Only 54 lawyers completed this survey limiting the generalizability of these results. However, these low response rates are consistent with other surveys examining lawyers’ attitudes or those working in the justice sector (Cox et al., 2008; McCormack et al., 2022). We were unable to recruit judges to participate in the survey, and further research is needed to understand how judges incorporate knowledge of FASD into their sentencing decisions The online format of this survey relied on participants’ choice to participate; participants with knowledge about FASD may have self-selected to take part in the survey and may be more knowledgeable than the general justice sector population. However, international research suggests that awareness of FASD is common amongst those in the justice sector (Cox et al., 2008; McCormack et al., 2022), consistent with the sample in this survey.

## Conclusion

Lack of knowledge and awareness of FASD is a significant barrier to fair and equitable treatment in the justice system and ensuring people have access to the support they need. Unless FASD is recognized and accommodated, many individuals with FASD will likely spend their lives revolving through the justice system, being misunderstood in court, victimized in prisons and mismanaged in the transition back to the community. This is a substantial financial burden and social cost to society (Fast & Conry, 2009) and leaves people with FASD vulnerable in the justice system.
